# Pre-stroke Functional Status in Patients Undergoing Mechanical Thrombectomy: How Relevant Are False Estimations in the Emergency Setting?

**DOI:** 10.1007/s00062-024-01449-5

**Published:** 2024-08-12

**Authors:** Marian Maximilian Hasl, João Pinho, Sophie Gina Baldus, Anna Gerhards, Martin Wiesmann, Jörg B. Schulz, Arno Reich, Omid Nikoubashman

**Affiliations:** 1 Department of Diagnostic and Interventional Neuroradiology, University Hospital, RWTH Aachen University, Aachen, Germany; 2 Department of Neurology, University Hospital, RWTH Aachen University, Pauwelsstr. 30, 52074 Aachen, Germany; 3 Forschungszentrum Jülich GmbH and RWTH Aachen University, JARA-BRAIN Institute Molecular Neuroscience and Neuroimaging, Aachen, Germany

**Keywords:** Stroke, Endovascular, Thrombectomy, Status, Functional

## Abstract

**Purpose:**

The modified Rankin scale (mRS) is frequently used in the emergency setting to estimate pre-stroke functional status in stroke patients who are candidates to acute revascularization therapies (ps-mRS). We aimed to describe the agreement between pre-stroke mRS evaluated in the emergency department (ED-ps-mRS) and pre-stroke mRS evaluated comprehensively post-admission (PA-ps-mRS).

**Methods:**

Retrospective study of consecutive ischemic stroke patients undergoing mechanical thrombectomy, with available ED-ps-mRS and PA-ps-mRS. ED-ps-mRS was evaluated by the treating neurologist and documented in the emergency stroke treatment protocol. PA-ps-mRS was retrospectively evaluated with information registered in the clinical record. Collection of baseline characteristics and 3‑month outcomes. Patients with ED-overestimated pre-stroke functional status (ED ps-mRS ≤ 2 and PA-ps-mRS ≥ 3) were compared to correct low and high ED-ps-mRS groups.

**Results:**

We included 409 patients (median age 77 years, 50% female, median NIHSS 14). Concordance of dichotomized ED-ps-mRS and PA-ps-mRS (0–2 vs. 3–5) was found in 81.4% (Cohen’s kappa = 0.476, *p* < 0.001). ED-overestimated pre-stroke functional status was found in 69 patients (17%). Patients with ED-overestimated pre-stroke functional status were older (*p* < 0.001), more frequently presented diabetes (*p* < 0.001), previous stroke (*p* = 0.014) and less frequently presented 3‑month functional independence (*p* < 0.001) compared to patients with correct low ED-ps-mRS. No differences in pre-stroke baseline characteristics between overestimated and correct high ED-ps-mRS was found.

**Conclusion:**

Disagreement between dichotomized ED-ps-mRS and PA-ps-mRS (0–2 vs. 3–5) occurred in 1/5 of patients. Overestimation of pre-stroke functional status may falsely reduce the expected proportion of patients achieving favourable 3‑month functional outcomes.

**Supplementary Information:**

The online version of this article (10.1007/s00062-024-01449-5) contains supplementary material, which is available to authorized users.

## Introduction

Stroke is the most common cause for permanent disability worldwide [1]. The extent of functional disability after stroke can be evaluated using the modified Rankin Scale (mRS), which is a clinician-reported 7‑level classification of global functional impairment [2]. mRS has been used in the majority of clinical trials studying acute treatment interventions in stroke patients, not only to define primary outcomes after stroke, but also to define pre-stroke functional disability as an exclusion criterion for many of these trials. Although mRS is a robust measure and is relatively easy to use, it has important limitations related to suboptimal interobserver reliability [3]. In daily clinical practice, pre-stroke functional status is an important information which allows clinicians to improve outcome prediction and helps them to advise patients and families on risks and benefits of available treatment interventions. In the emergency setting, pre-stroke mRS (ps-mRS) is used in many centres to estimate pre-stroke functional status in stroke patients who are candidates to acute revascularization therapies [4]. In the past decades, we have seen an increasing proportion of people who were already disabled and in need of care before they suffered a stroke [5]. Current international guidelines do not recommend to use pre-stroke disability as a sole criterion to withhold treatment with intravenous thrombolysis, however, absence of relevant pre-stroke disability, defined by mRS, is listed as an eligibility criterion to perform mechanical thrombectomy in acute stroke patients [6–8]. In clinical routine, these recommendations are often applied less strictly and research shows that patients with higher pre-stroke mRS can still benefit from mechanical thrombectomy [9]. Due to the emergency context, estimation of pre-stroke functional status before acute stroke treatment decisions is often based on insufficient information, not always obtained from an adequate source, and can lead to misclassification. This is problematic because an initial misclassification may result in a false estimation of outcome and may consequently wrongly influence important treatment decisions [10, 11]. There are some studies which identified absolute improvements of mRS 3 months after stroke compared to initial ps-mRS, which may indicate a poor interobserver reliability [9, 12, 13], and there is little evidence describing how ps-mRS misjudgement relates to functional outcome 3 months after stroke [11].

We aimed to describe the agreement between ps-mRS routinely evaluated in the emergency department (ED-ps-mRS) and ps-mRS evaluated comprehensively after admission (post-admission ps-mRS, PA-ps-mRS). Furthermore, we compared clinical and imaging characteristics, as well as treatment procedures in patients with correctly and incorrectly estimated ps-mRS.

## Methods

We conducted a monocentric, retrospective study of consecutive ischemic stroke patients undergoing mechanical thrombectomy included in our local prospective stroke registry, admitted in the period from February 2018 to March 2022. In our centre, acute stroke diagnostic workup, patient selection, thrombectomy technique and post-thrombectomy treatment follow the national and international recommendations. Pre-stroke functional disability, advanced age, and relevant co-morbidities are not considered contraindications for thrombectomy, and interdisciplinary treatment decisions are made on an individual basis.

### Data Collection

The following information was collected from the registry: demographic information, co-morbidities, clinical characteristics, baseline National Institutes of Health Stroke Scale (NIHSS), treatment with intravenous thrombolysis, achievement of successful recanalization (defined as a revised thrombolysis in cerebral infarction [TICI] score of 2b, 2c or 3) [14], cardioembolism as stroke etiology, occurrence of parenchymal hemorrhage type 2 (PH2) according to the definition of the European Cooperative Acute Stroke Study [15], in-hospital death, mRS at 3 months. Three-month outcome information is collected regularly for patients who are included in our local stroke registry in the setting of a hospital visit or telephone visit and is carried out by trained raters using a structured interview. Favourable 3‑month outcome was defined as a mRS of 0–2. ED-ps-mRS is routinely evaluated in the emergency department by the treating neurologist based on information provided by the patient or next of kin and is documented in the emergency stroke treatment protocol. For this evaluation we do not use in our centre a structured standardized interview, the information is collected as part of the routine clinical history. PA-ps-mRS was retrospectively evaluated by two of the investigators taking into consideration information documented in the clinical record of each patient by treating physicians in the stroke unit or neurointensive care unit, by the case management nurse team and by social service workers. Retrospective evaluation of PA-ps-mRS was blinded for outcome information. We assume that PA-ps-mRS is based on more reliable information and that it is, therefore, a more accurate assessment of pre-stroke functional status in comparison to ED-ps-mRS. Patients without available ED-ps-mRS and/or without sufficient information to estimate PA-ps-MRS were excluded from this study.

### Data Analysis

We divided our study population into four subgroups based on the agreement between ED-ps-mRS and PA-ps-mRS: ED-overestimated pre-stroke functional status (ED-ps-mRS ≤ 2 and PA-ps-mRS ≥ 3), ED-underestimated pre-stroke functional status (ED-ps-mRS ≥ 3 and PA-ps-mRS ≤ 2), correct low ED-ps-mRS (both ED-ps-mRS and PA-ps-mRS ≤ 2); correct high ED-ps-mRS (both ED-ps-mRS and PA-ps-mRS ≥ 3). The choice for this categorization of mRS using the limit ≤ 2 vs. ≥ 3 was based on the fact that these two groups represent patients who have functional independence (mRS ≤ 2) or patients who need help to carry out their daily life activities (mRS ≥ 3). Cohens Kappa (κ) was used to assess interobserver agreement between ED-ps-mRS and PA-ps-mRS, for both absolute agreement for each mRS level and for dichotomous agreement (ps-mRS 0–2 vs. ps-mRS 3–5). We compared patients with overestimated pre-stroke functional status with patients with a correct high ED-ps-mRS, and with patients with a correct low ED-ps-mRS. Data distribution was tested using the Shapiro-Wilk test. Univariate analyses were performed using the Chi-square test for categorical variables, Mann-Whitney‑U test was used for continuous variables without normal distribution. We analysed independent baseline predictors for ED overestimation of pre-stroke functional status (versus patients with correct ED estimation of pre-stroke functional status) by using a multivariable binary logistic regression analysis to calculate the unadjusted odds ratio (OR) and the 95% confidence intervals (95%CI). Baseline variables significantly associated with an ED overestimation of pre-stroke functional status in comparison to correct low ED-ps-mRS in the univariable analyses were included in the model as covariates.

We conducted sensitivity analyses by grouping our study population according to a lower mRS score limit: ED-overestimated pre-stroke functional status (ED-ps-mRS ≤ 1 and PA-ps-mRS ≥ 2), ED-underestimated pre-stroke functional status (ED-ps-mRS ≥ 2 and PA-ps-mRS ≤ 1), correct low ED-ps-mRS (both ED-ps-mRS and PA-ps-mRS ≤ 1); correct high ED-ps-mRS (both ED-ps-mRS and PA-ps-mRS ≥ 2). We conducted further sensitivity analyses by grouping our study population according to the presence or absence of ED-overestimated pre-stroke functional status defined as PA-ps-mRS higher than ED-ps-mRS.

An alpha level of less than 0.05 was considered statistically significant. Statistical analyses were performed with IBM SPSS Statistics (Version 28.0.1.0). No missing data imputation was performed. This report follows the Strengthening the Reporting of Observational Studies in Epidemiology (STROBE) guidelines [16]. Retrospective studies based on the local registry of patients with acute ischemic strokes were approved by the local ethics committee (approval reference 335/15) and, because of the retrospective nature, the ethics committee waived the need for patient-signed consent.

## Results

During the 4‑year study period, a total of 787 acute ischemic stroke patients underwent mechanical thrombectomy. We excluded a total of 378 patients from this study: 306 patients did not have available ED-ps-mRS, 36 patients did not have sufficient documented information to estimate PA-ps-mRS, and 36 did not have both. Our final study population comprised 409 patients, with a median age of 77 years (interquartile range (IQR) 65.4–84.1), 205 were female (50.1%). Median NIHSS at admission was 14 (IQR 8–18), 178 patients (43.5%) received intravenous thrombolysis and 379 (92.7%) had a successful recanalization. Three-month mRS was available for 310 patients, 82 (26.5%) of whom achieved a favourable 3‑month outcome.

**Fig. 1:**
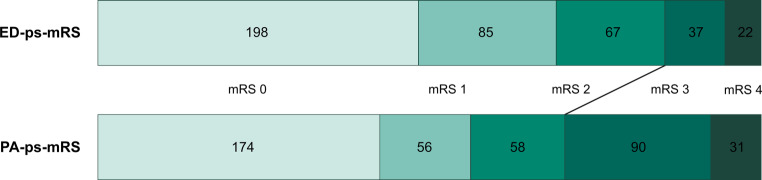
Distribution of emergency department pre-stroke modified Rankin Scale (ED-ps-mRS) and post-admission pre-stroke modified Rankin Scale (PA-ps-mRS)

Distribution of ED-ps-mRS and PA-ps-mRS is presented in Fig. 1.

Absolute concordance between ED-ps-mRS and PA-ps-mRS was found in 272 patients (66.5%). Dichotomous concordance (ps-mRS 0–2 vs. ps-mRS 3–5) was found in 333 patients (81.4%). Absolute median difference between PA-ps-mRS and ED-ps-mRS was 0 (IQR 0–1). Interobserver agreement was moderate for both absolute agreement (κ = 0.535, *p* < 0.001) and for dichotomous agreement (κ = 0.476, *p* < 0.001). ED-overestimated pre-stroke functional status was found in 69 patients (16.9%), ED-underestimated pre-stroke functional status was found in 7 patients (1.7%), correct low ED-ps-mRS was found in 281 patients (68.7%), and correct high ED-ps-mRS was found in 52 patients (12.7%). Distribution of 3‑month outcome according to ps-mRS assessed in the emergency department and post-admission is presented in Supplementary Fig. 1.

### ED-overestimated Pre-stroke Functional Status Versus Correct Low ED-ps-mRS

Compared to patients with a correct low ED-ps-mRS, patients with ED-overestimated pre-stroke functional status were older (83.1 vs. 72.3 years, *p* < 0.001) and more often female (65.2% vs. 45.2%, *p* = 0.003), more frequently had diabetes mellitus (43.5% vs. 17.8%, *p* < 0.001), atrial fibrillation (60.9% vs. 37.0%, *p* < 0.001), and previous stroke (29.0% vs. 13.5%, *p* = 0.002) (Table 1). Patients with ED-overestimated pre-stroke functional status underwent intravenous thrombolysis less frequently (30.4% vs. 47.7%, *p* = 0.010), achieved a successful recanalization more often (100% vs. 91.1%, *p* = 0.010) and had a higher prevalence of cardioembolic aetiology (69.1% vs. 53.0%, *p* = 0.016). No patient with an overestimated pre-stroke functional status achieved functional independence at 3 months (0% vs. 38.3%, *p* < 0.001).

**Table 1:** Characteristics of the study population according to grouping in overestimation of pre-stroke functional status (emergency department pre-stroke modified Rankin Scale ≤ 2 and post-admission pre-stroke modified Rankin Scale ≥ 3), correct low emergency department pre-stroke modified Rankin Scale and correct high emergency department pre-stroke modified Rankin Scale

	ED-overestimated pre-stroke functional status (*n* = 69)	Correct low ED-ps-mRS (*n* = 281)	*p*	Correct high ED-ps-mRS (*n* = 52)	p*
Age (years)	83.1 (77.7–87.9)	72.3 (63.2–80.8)	< 0.001	82.8 (77.4–90.2)	0.738
Female sex	45 (65.2)	127 (45.2)	0.003	29 (55.8)	0.291
Arterial hypertension	58 (84.1)	216 (76.9)	0.194	47 (90.4)	0.309
Diabetes mellitus	30 (43.5)	50 (17.8)	< 0.001	20 (38.5)	0.579
Dyslipidemia	21 (30.4)	79 (28.1)	0.702	15 (28.8)	0.850
Current or past smoker	12 (17.4)	68 (24.2)	0.228	5 (9.6)	0.223
Atrial fibrillation	42 (60.9)	104 (37.0)	< 0.001	31 (59.6)	0.889
Previous ischemic stroke	20 (29.0)	38 (13.5)	0.002	17 (32.7)	0.661
Baseline NIHSS^§^	15 (9–19)	14 (8–18)	0.483	15 (10–18)	0.624
Intravenous thrombolysis	21 (30.4)	134 (47.7)	0.010	20 (38.5)	0.356
Successful recanalization	69 (100)	256 (91.1)	0.010	47 (90.4)	0.009
Cardioembolic etiology^‡^	47 (69.1)	149 (53.0)	0.016	39 (75.0)	0.479
Parenchymal hemorrhage type 2	0 (0.0)	7 (2.5)	0.185	4 (7.7)	0.019
In-hospital death	17 (24.6)	65 (23.1)	0.791	28 (53.8)	< 0.001
Functional independence at 3 months (mRS 0–2)^†^	0 (0.0)	80 (38.3)	< 0.001	0 (0.0)	–
Death at 3 months^†^	24 (48.0)	71 (34.0)	0.064	34 (73.9)	0.009

*ED-ps-mRS* emergency department pre-stroke modified Rankin Scale, *NIHSS* National Institutes of Health Stroke Scale.

* Refers to comparison between patients with overestimated pre-stroke functional status and patients with correct high ED-ps-mRS

^§^ Missing information for 3 patients

^‡^ Missing information for 1 patient

^†^ 3‑month functional status available for 259 patients

The sensitivity analysis with grouping of patients according to a lower mRS score (≤ 1 vs. ≥ 2) did not change the results significantly, except that previous ischemic stroke and intravenous thrombolysis were not different in the two groups (Supplementary Table 1).

When we defined ED-overestimated pre-stroke functional status as PA-ps-mRS higher than ED-ps-mRS (absolute difference ≥ 1), presence of ED-overestimation of pre-stroke functional status was associated with older age, female sex, diabetes mellitus, atrial fibrillation, successful recanalization, cardioembolic etiology, and lower frequency of 3‑month functional independence (Supplementary Table 2).

### ED-overestimated Pre-stroke Functional Status Versus Correct High ED-ps-mRS

When compared to patients with a correct high ED-ps-mRS, pre-stroke baseline characteristics of patients with ED-overestimated pre-stroke functional status did not differ significantly (Table 1). Patients with ED-overestimated pre-stroke functional status achieved a successful recanalization more frequently (100% vs. 90.4%, *p* = 0.009), developed a parenchymal hemorrhage type 2 less frequently (0% vs. 7.7%, *p* = 0.019) and had lower in-hospital and 3‑month mortality (24.6% vs. 53.8%, *p* < 0.001 and 48.0% vs. 73.9%, *p* = 0.009, respectively). The sensitivity analysis with grouping of patients according to a lower mRS score (≤ 1 vs. ≥ 2) did not change the results significantly (Supplementary Table 3).

### Baseline Predictors for ED Overestimation of Pre-stroke Functional Status

The multivariable logistic regression analysis (Table 2) revealed that the only independent baseline predictors for ED overestimation of pre-stroke functional status were older age (OR per year increase = 1.07, 95%CI = 1.03–1.10, *p* < 0.001), presence of diabetes (OR = 2.91, 95%CI = 1.63–5.17, *p* < 0.001) and previous stroke (OR = 2.23, 95%CI = 1.18–4.24, *p* = 0.014). The sensitivity analysis with grouping of patients according to a lower mRS score (≤ 1 vs. ≥ 2) revealed that increasing age was the only factor independently associated with ED overestimation of pre-stroke functional status (Supplementary Table 4).

**Table 2:** Multivariable binary logistic regression model for baseline prediction of emergency department overestimation of pre-stroke functional status (versus patients with correct emergency department pre-stroke modified Rankin Scale)

	Odds ratio (95% confidence interval)	*p*
Age (per 1‑year increase)	1.07 (1.03–1.10)	< 0.001
Female sex	1.61 (0.90–2.90)	0.612
Diabetes mellitus	2.91 (1.63–5.17)	< 0.001
Atrial fibrillation	1.37 (0.76–2.47)	0.290
Previous ischemic stroke	2.23 (1.18–4.24)	0.014

## Discussion

In this study we analysed the agreement between ps-mRS evaluated in the emergency department (ED-ps-mRS) and evaluated in more detail after admission (PA-ps-mRS). Disagreement between ED-ps-mRS and PA-ps-mRS dichotomized into 0–2 and 3–5, occurred in one-fifth of the patients. Absolute agreement and dichotomous agreement for the two assessments were moderate. For the majority of patients in whom disagreement was observed, an overestimation of pre-stroke functional status occurred (i.e. a better pre-stroke functional status was estimated, in comparison to the real pre-stroke functional status). These findings are relevant for real life clinical practice, because they show that in a significant proportion of stroke patients undergoing mechanical thrombectomy, treatment decisions are based on false information concerning pre-stroke functional status. This may induce inaccurate counselling of the patients and their care persons in the acute setting and create higher expectations both for clinicians and patients concerning treatment benefit. A typical example of this is the case of a patient with M1 occlusion presenting within the 6‑hour time window, with a pre-stroke mRS of 3 (functional dependence, ability to walk unassisted), which was falsely estimated as 2 (functional independence, ability to walk unassisted) in the emergency department. According to the current evidence and taking into consideration the falsely estimated ps-mRS of 2, the chance of this patient of achieving a good 3‑month outcome (functional independence) with mechanical thrombectomy would be 24% [17]. However, when taking into consideration the correct ps-mRS of 3, one would expect only an 8% chance of achieving a good 3‑month outcome (functional independence) [9].

The reasons for disagreement between the two pre-stroke functional status assessments are complex. They probably include the fact that a significant proportion of patients is not able to provide reliable information due to neurological impairment, the need for a rapid evaluation in the emergency setting which does not allow a detailed evaluation with an adequate family member or next of kin, cases of withholding information pointing to disability by patients or next of kin with the goal of avoiding therapy limitation, and tendency to overlook information pointing to disability by the treating physicians with the goal of providing maximal state-of-the-art therapy to as many patients as possible. Overestimating pre-stroke functional status in daily clinical practice has the immediate implication of increasing the proportion of stroke patients treated with advanced invasive therapies such as mechanical thrombectomy, but it also sets a higher expectation for clinicians, patients and families for the extent of the clinical benefit of acute stroke treatments.

The majority of randomized clinical trials studying the benefit of endovascular treatment in stroke patients with large vessel occlusion did not include patients with relevant pre-stroke disability, which was defined either by modified Rankin scale greater as 1 or 2, or by Barthel index lower than 90 points [18, 19]. An exception to this was the MR CLEAN trial, which included 4% of patients with pre-stroke functional dependence [20]. However, many clinicians consider performing mechanical thrombectomy even in stroke patients with pre-existing disability [21]. Although ischemic stroke patients with pre-existing disability present overall worse functional outcome [22], there is still a beneficial treatment effect of mechanical thrombectomy [17]. The main implication of overestimating pre-stroke functional status in stroke research is that the proportion of patients achieving favourable outcomes (3-month mRS 0–2) will be falsely lower than expected, because these patients will rarely be able to improve from their real pre-stroke functional status.

In a previous study of consecutive large vessel occlusion stroke patients undergoing mechanical thrombectomy, Prakapenia and collaborators found misjudgement of pre-stroke functional status using mRS in a significant proportion of patients (34%), and discuss that the validity of mRS appears to be low in acute stroke settings [11]. These findings appear to challenge the results of a prospective cohort study of stroke patients by Quinn and collaborators, where ps-mRS was indeed significantly associated with the Charlson co-morbidity index and pre-stroke living conditions [10]. Another prospective study conducted in stroke units concluded that the validity of ps-mRS was suboptimal, but also found significant associations between ps-mRS and pre-stroke comorbidity and pre-stroke frailty [23]. However, the main differences to our study and to the study of Prakapenia and collaborators, is that, in the studies by Quinn [10] and Fearon [23], ps-mRS was not evaluated in the context of the emergent care before decisions related to acute revascularisation therapies.

We found that the only independent baseline predictors of overestimating pre-stroke functional status were older age, presence of diabetes mellitus and previous stroke. In the study by Prakapenia and collaborators, patients with ps-mRS misjudgement were older, and more frequently had diabetes mellitus, as well as atrial fibrillation, but no multivariable analysis was conducted [11]. Clinicians should be aware that false estimations of pre-stroke functional status are more frequent in older patients and in patients with diabetes and previous stroke, and should perform a more detailed assessment in this subgroup of patients. The use of standardized validated mRS questionnaires, which are of simple application and not time-consuming [24], may also provide increased validity in comparison to an unstructured assessment.

Another important finding of our study is that patients with correct high ED-ps-mRS and patients with ED-overestimated pre-stroke functional status did not differ in their pre-stroke baseline characteristics, which supports the validity of our post-admission assessment ps-mRS. This is explained by their overall poor pre-stroke functional status, indicating that they indeed belong to the same group and were misclassified. However, the significantly higher mortality in the group of correct high ED-ps-mRS suggests that other baseline characteristics, including other co-morbidities or other robust clinical markers for pre-stroke disability not encompassed by our study, may come into play in the assessment of pre-existing disabilities, and mediate the increased mortality. We cannot exclude the possibility that treatment strategies and decisions concerning therapy limitations after mechanical thrombectomy differed between these two groups of patients, and that this could have influenced the observed difference in mortality.

The main limitations of our study include the retrospective and monocentric nature of the study. We excluded a significant number of patients for whom no assessment of pre-stroke functional status in the ED was available, which may induce relevant selection bias, and the analysis is also limited by missing 3‑month outcome information. We dichotomized ps-mRS into categories which are clinically relevant (0–2 and 3–5), and therefore tried to minimize the impact of bias resulting from suboptimal validity of mRS and variability of subjective assessment. One of the main strengths of our study is that a comprehensive multidisciplinary assessment of pre-stroke functional status was used to classify PA-ps-mRS.

## Conclusion

Agreement between the assessment of pre-stroke functional status in the emergency department and a detailed assessment after hospital admission is moderate. An incorrect assessment of pre-stroke functional status in the emergency department occurs in one-fifth of all acute ischemic stroke patients undergoing mechanical thrombectomy. Older age, diabetes mellitus and previous stroke are independent predictors of overestimation of pre-stroke functional status in the emergency department. The correct assessment of pre-stroke functional status in the emergency department is challenging, but, at the same time, it is very important to support adequate treatment decisions and valid outcome predictions.

## Supplementary Information

Supplementary information contains a supplementary figure illustrating the relationship between pre-stroke modified Rankin scale and 3-month modified Rankin scale, and the results of sensitivity analyses.

## Data Availability

Data will be made available upon reasonable request to the corresponding author, for research purposes and according to the local recommendations.
