# Supplementary material for: Pre-stroke Functional Status in Patients Undergoing Mechanical Thrombectomy: How Relevant Are False Estimations in the Emergency Setting?
Source: Clin Neuroradiol. 2024 Aug 12;35(1):17–23. doi: 10.1007/s00062-024-01449-5 (PMC11832557; doi:10.1007/s00062-024-01449-5)
Supplement: Supplementary file 1 — Supplementary information contains a supplementary figure illustrating the relationship between pre-stroke modified Rankin scale and 3-month modified Rankin scale, and the results of sensitivity analyses. [file 62_2024_1449_MOESM1_ESM.docx]

**Supplementary Material**

**Supplementary Figure 1.** Alluvial diagram illustrating the relationship between emergency department pre-stroke modified Rankin scale (mRS) and post-admission pre-stroke mRS with mRS at 3-months in the 310 patients with known 3-month outcome.


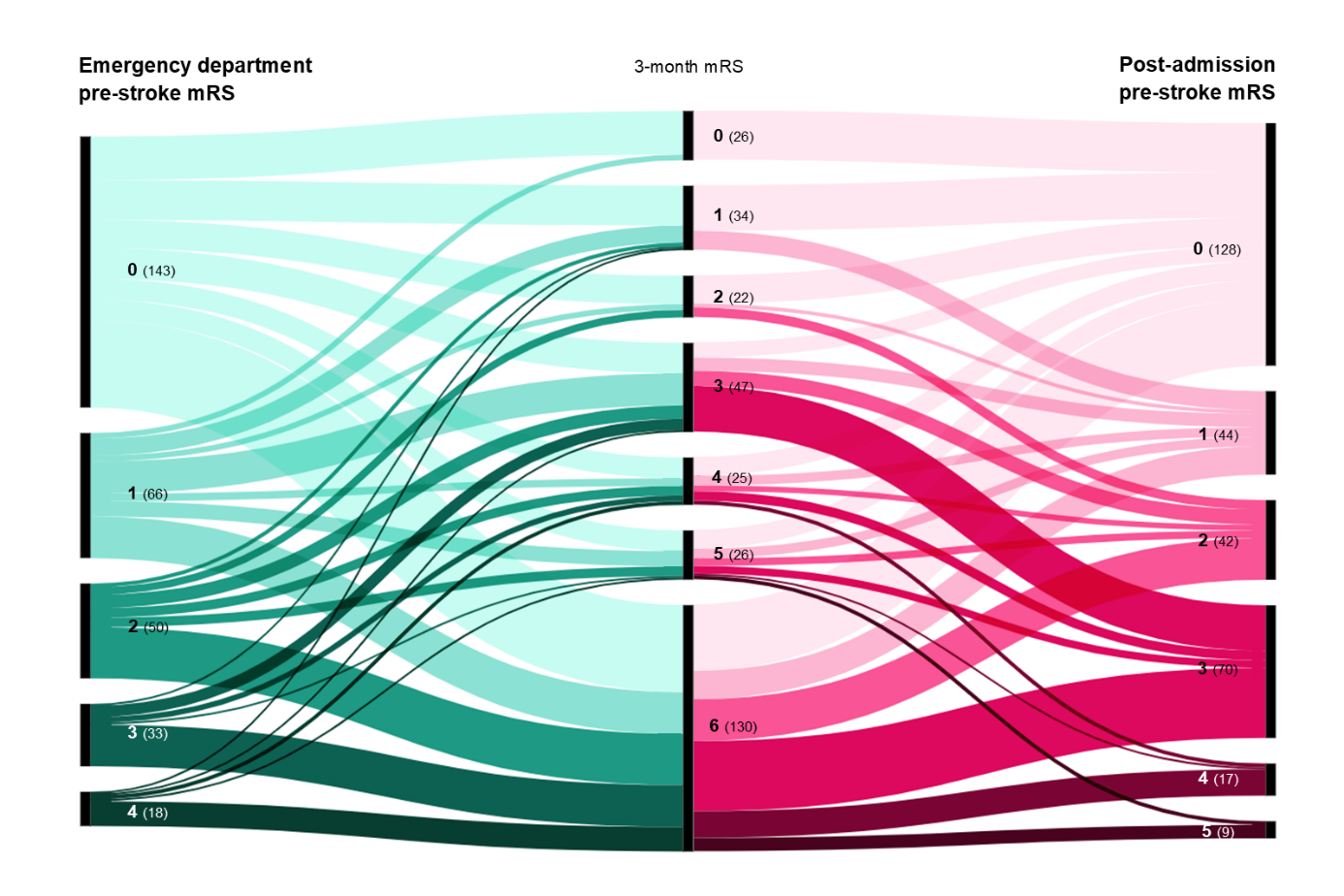


**Supplementary Table 1.** Comparison of the characteristics of final study population according to grouping in correct low emergency department pre-stroke modified Rankin Scale and overestimation of pre-stroke functional status (emergency department pre-stroke modified Rankin Scale ≤1 and post-admission pre-stroke modified Rankin Scale ≥2)

|  | **Correct low ED-ps-mRS (n=218)** | **ED overestimated pre-stroke functional status (n=65)** | **p** |
| --- | --- | --- | --- |
| Age (years) | 71.3 (62.6-80.2) | 82.7 (73.5-86.9) | <0.001 |
| Female sex | 92 (42.2) | 41 (63.1) | 0.003 |
| Arterial hypertension | 161 (73.9) | 54 (83.1) | 0.127 |
| Diabetes mellitus | 34 (15.6) | 21 (32.3) | 0.003 |
| Dyslipidemia | 60 (27.5) | 18 (27.7) | 0.979 |
| Current or past smoker | 51 (23.4) | 8 (12.3) | 0.053 |
| Atrial fibrillation | 73 (33.5) | 38 (58.5) | <0.001 |
| Previous ischemic stroke | 30 (13.8) | 12 (18.5) | 0.350 |
| Baseline NIHSS* | 14 (8-18) | 15 (9-18) | 0.455 |
| Intravenous thrombolysis | 112 (51.4) | 26 (40.0) | 0.107 |
| Successful recanalization | 198 (90.8) | 64 (98.5) | 0.039 |
| Cardioembolic etiology* | 111 (50.9) | 44 (68.8) | 0.012 |
| Parenchymal hemorrhage type 2 | 7 (3.2) | 0 (0.0) | 0.143 |
| In-hospital death | 41 (18.8) | 16 (24.6) | 0.305 |
| Functional independence at 3 months (mRS 0-2)^†^ | 56 (34.8) | 0 (0.0) | <0.001 |
| Death at 3 months^†^ | 46 (28.6) | 22 (45.8) | 0.025 |

ED-ps-mRS: emergency department pre-stroke modified Rankin Scale. NIHSS: National Institutes of Health Stroke Scale.

* Missing information for 1 patient

^†^ 3-month functional status available for 209 patients

**Supplementary Table 2.** Comparison of the characteristics of final study population according to the presence or absence of ED-overestimated pre-stroke functional status, defined as PA-ps-mRS higher than ED-ps-mRS (absolute difference ≥1).

|  | **No ED-overestimation of functional status (n=301)** | **ED-overestimation of functional status (n=108)** | **p** |
| --- | --- | --- | --- |
| Age (years) | 74.0 (63.7-81.4) | 82.8 (73.1-87.2) | <0.001 |
| Female sex | 138 (45.8) | 67 (62.0) | 0.004 |
| Arterial hypertension | 235 (78.1) | 93 (86.1) | 0.072 |
| Diabetes mellitus | 60 (19.9) | 41 (38.0) | <0.001 |
| Dyslipidemia | 86 (28.6) | 29 (26.9) | 0.733 |
| Current or past smoker | 67 (22.3) | 19 (17.6) | 0.307 |
| Atrial fibrillation | 120 (39.9) | 62 (57.4) | 0.002 |
| Previous ischemic stroke | 52 (17.3) | 24 (22.2) | 0.257 |
| Baseline NIHSS* | 14 (8-18) | 15 (9-19) | 0.215 |
| Intravenous thrombolysis | 134 (44.5) | 44 (40.7) | 0.497 |
| Successful recanalization | 274 (91.0) | 105 (97.2) | 0.034 |
| Cardioembolic etiology^‡^ | 165 (54.8) | 75 (70.1) | 0.006 |
| Parenchymal hemorrhage type 2 | 9 (3.0) | 2 (1.9) | 0.531 |
| In-hospital death | 80 (26.6) | 31 (28.7) | 0.670 |
| Functional independence at 3 months (mRS 0-2)^†^ | 78 (34.2) | 4 (4.9) | <0.001 |
| Death at 3 months^†^ | 91 (39.9) | 39 (47.6) | 0.229 |

PA-ps-mRS: post-admission pre-stroke modified Rankin Scale. ED-ps-mRS: emergency department pre-stroke modified Rankin Scale. NIHSS: National Institutes of Health Stroke Scale.

* Missing information for 3 patients

^‡^ Missing information for 2 patients

^†^ 3-month functional status available for 310 patients

**Supplementary Table 3.** Comparison of the characteristics of final study population according to grouping in correct high emergency department pre-stroke modified Rankin Scale and overestimation of pre-stroke functional status (emergency department pre-stroke modified Rankin Scale ≤1 and post-admission pre-stroke modified Rankin Scale ≥2)

|  | **Correct high ED-ps-mRS (n=114)** | **ED overestimated pre-stroke functional status (n=65)** | **p** |
| --- | --- | --- | --- |
| Age (years) | 81.0 (72.8-88.5) | 82.7 (73.5-86.9) | 0.794 |
| Female sex | 66 (57.9) | 41 (63.1) | 0.497 |
| Arterial hypertension | 103 (90.4) | 54 (83.1) | 0.154 |
| Diabetes mellitus | 43 (37.7) | 21 (32.3) | 0.468 |
| Dyslipidemia | 35 (30.7) | 18 (27.7) | 0.671 |
| Current or past smoker | 24 (21.1) | 8 (12.3) | 0.142 |
| Atrial fibrillation | 69 (60.5) | 38 (58.5) | 0.786 |
| Previous ischemic stroke | 33 (28.9) | 12 (18.5) | 0.120 |
| Baseline NIHSS* | 15 (9-19) | 15 (9-18) | 0.850 |
| Intravenous thrombolysis | 38 (33.3) | 26 (40.0) | 0.371 |
| Successful recanalization | 106 (93.0) | 64 (98.5) | 0.107 |
| Cardioembolic etiology^‡^ | 80 (70.2) | 44 (68.8) | 0.843 |
| Parenchymal hemorrhage type 2 | 4 (3.5) | 0 (0.0) | 0.127 |
| In-hospital death | 50 (43.9) | 16 (24.6) | 0.010 |
| Functional independence at 3 months (mRS 0-2)^†^ | 0 (0.0) | 0 (0.0) | - |
| Death at 3 months^†^ | 58 (64.4) | 22 (45.8) | 0.035 |

ED-ps-mRS: emergency department pre-stroke modified Rankin Scale. NIHSS: National Institutes of Health Stroke Scale.

* Missing information for 2 patients

^‡^ Missing information for 1 patient

^†^ 3-month functional status available for 138 patients

**Supplementary Table 4.** Multivariable binary logistic regression model for baseline prediction of emergency department overestimation of pre-stroke functional status (emergency department pre-stroke modified Rankin Scale ≤1 and post-admission pre-stroke modified Rankin Scale ≥2) (versus patients with correct emergency department pre-stroke modified Rankin Scale)

|  | **Odds ratio (95% confidence interval)** | **p** |
| --- | --- | --- |
| Age (per 1-year increase) | 1.04 (1.01-1.07) | 0.005 |
| Female sex | 1.40 (0.78-2.49) | 0.259 |
| Diabetes mellitus | 1.56 (0.86-2.82) | 0.143 |
| Atrial fibrillation | 1.29 (0.72-2.30) | 0.398 |
| Previous ischemic stroke | 1.00 (0.50-2.02) | 0.994 |
